# Supplementary material for: Ret function in muscle stem cells points to tyrosine kinase inhibitor therapy for facioscapulohumeral muscular dystrophy
Source: eLife. 2016 Nov 14;5:e11405. doi: 10.7554/eLife.11405 (PMC5108591; doi:10.7554/eLife.11405)
Supplement: Figure 7—source data 1. — (a) Maximum likelihood parameters for a logistic model containing an interaction term, and a random effect term (the mouse) describing the probability of a nucleus being present in a MyHC+ cells. (b) Corresponding ratios computed from the model, for all 4 tested conditions with the ratio representing the probability of a GFP+ cell expressing MyHC. In all condition but the control (Intercept), the error contribution of the baseline (Intercept) has been omitted when computing the confidence intervals (C.I.). y represents the log-of-odds of the fusion index. µ represents the intercept parameter (representing the control: MIG control retrovirus, control siRNA), β are the parameters representing the effects of each treatment, or the interaction as specified and δ indicates whether the effect is present or absent. DOI: http://dx.doi.org/10.7554/eLife.11405.009 [file elife-11405-fig7-data1.docx]

**Figure 7: Supplementary Table**

(a) Maximum likelihood parameters for a logistic model containing an interaction term, and a random effect term (the mouse) describing the probability of a nucleus being present in a MyHC+ cells. (b) Corresponding ratios computed from the model, for all 4 tested conditions with the ratio representing the probability of a GFP+ cell expressing MyHC. In all condition but the control (Intercept), the error contribution of the baseline (Intercept) has been omitted when computing the confidence intervals (C.I.). *y* represents the log-of-odds of the fusion index. µ represents the intercept parameter (representing the control: MIG control retrovirus, control siRNA), *β* are the parameters representing the effects of each treatment, or the interaction as specified and δ indicates whether the effect is present or absent.

a)

Parameter   Estimate  Std.err.   t value   P value

Intercept     -0.208     0.177    -1.174     0.240

DUX4          -1.456     0.085   -17.064   1.7 x 10-65

RET            0.164     0.045     3.610   0.00031

Interaction    0.970     0.102     9.498   2.1 x 10-21

b)

Treatment      Ratio  Low C.I. High C.I.

control+MIG    0.448     0.365     0.535

control+DUX4   0.159     0.138     0.183

RET+MIG        0.489     0.447     0.512

RET+DUX4       0.371     0.344     0.398
