# Supplementary material for: Ret function in muscle stem cells points to tyrosine kinase inhibitor therapy for facioscapulohumeral muscular dystrophy
Source: eLife. 2016 Nov 14;5:e11405. doi: 10.7554/eLife.11405 (PMC5108591; doi:10.7554/eLife.11405)
Supplement: Figure 8—source data 1. — (a) Maximum likelihood parameters for a logistic model containing an interaction term and a random effect (mouse) to describe the effect of RET51-MENA expression and Sunitinib, TG101209 or Zactima on fusion in C2C12 myoblasts. (b) Maximum likelihood parameters for a logistic model containing an interaction term for RET51-MEN2A and Sunitinib (RET51CA:Sunitinib), Ret51-MEN2A with TG101209 (RET51CA: TG101209) or Ret5-MEN2A with Zactima (RET51CA:Zactima) that reveals the gradient of response of myoblast fusion. Significance of interaction effects relative to the baseline (MIG control retrovirus infected cells with no drug present) is indicated by p values. y represents the log of the odds of the fusion index, µ represents the intercept parameter (representing the control treatment: MIG control retrovirus with no drug), β are the parameters representing the effects of each treatment, or the interaction as specified, c is a parameter representing the effect of concentration of a drug and δ indicates whether the effect is present or absent. DOI: http://dx.doi.org/10.7554/eLife.11405.011 [file elife-11405-fig8-data1.docx]

**Figure 8: Supplementary Table 1**

(a) Maximum likelihood parameters for a logistic model containing an interaction term and a random effect (mouse) to describe the effect of RET51-MENA expression and Sunitinib, TG101209 or ZACTIMA on fusion in C2C12 myoblasts. (b) Maximum likelihood parameters for a logistic model containing an interaction term for RET51-MEN2A and Sunitinib (RET51CA:Sunitinib), Ret51-MEN2A with TG101209 (RET51CA: TG101209) or Ret5-MEN2A with ZACTIMA (RET51CA:Zactima) that reveals the gradient of response of muscle fusion. Significance of interaction effects relative to the baseline (MIG control retrovirus infected cells with no drug present) is indicated by P values. *y* represents the log of odds of the fusion index, µ represents the intercept parameter (representing the control treatment: MIG control retrovirus with no drug), *β* are the parameters representing the effects of each treatment, or the interaction as specified, *c* is a parameter representing the effect of concentration of a drug and δ indicates whether the effect is present or absent.

a)

Parameter                  Estimate Std. Error z value Pr(>|z|)

(Intercept)                 0.02587    0.30368   0.09    0.932

Sunitinib 0.25             0.08553    0.03942   2.17    0.030 *

Sunitinib 0.5              0.15497    0.03829   4.05 5.18e-05 ***

Sunitinib 1                0.30173    0.04066   7.42 1.16e-13 ***

TG101209 0.025             -0.03234    0.03567   -0.91    0.365

TG101209 0.05              -0.34706    0.03680   -9.43  < 2e-16 ***

TG101209 0.1               -0.21284    0.03827   -5.56 2.67e-08 ***

Zactima 0.25               -0.20144    0.04130   -4.88 1.07e-06 ***

Zactima 0.5                -0.01508    0.03806   -0.40    0.692

Zactima 1                  -0.42687    0.04698   -9.09  < 2e-16 ***

RET51CA               -3.34058    0.05256  -63.56  < 2e-16 ***

Sunitinib 0.25:RET51CA 0.97212    0.07414  13.11  < 2e-16 ***

Sunitinib 0.5:RET51CA  2.21183    0.06574  33.65  < 2e-16 ***

Sunitinib 1:RET51CA    2.30204    0.06745  34.13  < 2e-16 ***

TG101209 0.025:RET51CA 1.42148    0.06745  21.07  < 2e-16 ***

TG101209 0.05:RET51CA  1.95364    0.06611  29.55  < 2e-16 ***

TG101209 0.1:RET51CA   1.46939    0.07126  20.62  < 2e-16 ***

Zactima 0.25:RET51CA   2.83161    0.07073  40.03  < 2e-16 ***

Zactima 0.5:RET51CA    2.98206    0.06897  43.23  < 2e-16 ***

Zactima 1:RET51CA      3.94622    0.07941  49.69  < 2e-16 ***

b)

Parameter             Estimate Std. Error z value Pr(>|z|)

(Intercept)            0.008026   0.308986   0.03    0.979

RET51CA            -2.157703   0.024436  -88.30  < 2e-16 ***

Sunitinib 0.326147   0.036056   9.05  < 2e-16 ***

RET51CA:Sunitinib  1.574679   0.032506  48.44  < 2e-16 ***

TG101209   -2.873471   0.342331   -8.39  < 2e-16 ***

RET51CA:TG101209   3.295843   0.386403   8.53  < 2e-16 ***

Zactima    -0.304531   0.040307   -7.56 4.18e-14 ***

RET51CA:Zactima    2.923664   0.045197  64.69  < 2e-16 ***
