# Supplementary material for: Ret function in muscle stem cells points to tyrosine kinase inhibitor therapy for facioscapulohumeral muscular dystrophy
Source: eLife. 2016 Nov 14;5:e11405. doi: 10.7554/eLife.11405 (PMC5108591; doi:10.7554/eLife.11405)
Supplement: Figure 8—source data 2. — The significance of each effect and combined effects relative to the baseline (control (MIG) infected cells from Batch A with no drug present) is indicated by p values. y represents the log of the number of cells, µ represents the intercept parameter (representing the control treatment: MIG control retrovirus with no drug), β are the parameters representing the effects of each treatment, or the interaction as specified and δ indicates whether the effect is present or absent. DOI: http://dx.doi.org/10.7554/eLife.11405.012 [file elife-11405-fig8-data2.docx]

**Figure 8: Supplementary Table 2**

Quasi-Poisson model parameters for a fixed-effects factorial model incorporating a parameter to account for replicate effects (Batch) on the number of cells expressing RET51-MEN2A (RET51CA) or MIG (no label) when treated with different concentrations of Sunitinib, TG101209 or ZACTIMA. Significance of each effect and combined effects relative to the baseline (MIG infected cells from Batch A with no drug present) is indicated by P values. *y* represents the log of the number of cells, µ represents the intercept parameter (representing the control treatment: MIG control retrovirus with no drug), *β* are the parameters representing the effects of each treatment, or the interaction as specified and δ indicates whether the effect is present or absent.

Parameter                Estimate Std. Error t value Pr(>|t|)

(Intercept)                 5.42864    0.08072  67.250  < 2e-16 ***

Sunitinib 0.25              0.03437    0.12384   0.278  0.78159

Sunitinib 0.5               0.13431    0.11990   1.120  0.26375

Sunitinib 1                -0.01669    0.12596  -0.133  0.89469

TG101209 0.025              0.34541    0.11241   3.073  0.00236 **

TG101209 0.05               0.23485    0.11619   2.021  0.04437 *

TG101209 0.1                0.10314    0.12110   0.852  0.39522

Zactima 0.25               -0.01365    0.13024  -0.105  0.91663

Zactima 0.5                -0.03546    0.11965  -0.296  0.76718

Zactima 1                  -0.43784    0.14641  -2.990  0.00307 **

RET51CA                     0.39868    0.09349   4.264 2.88e-05 ***

BatchB                      0.52266    0.05160  10.129  < 2e-16 ***

BatchC                      0.54335    0.05110  10.632  < 2e-16 ***

Sunitinib 0.25:RET51CA   0.10271    0.16148   0.636  0.52536

Sunitinib 0.5:RET51CA   0.09165    0.15326   0.598  0.55037

Sunitinib 1:RET51CA     0.08677    0.16101   0.539  0.59044

TG101209 0.025:RET51CA -0.09561    0.14702  -0.650  0.51611

TG101209 0.05:RET51CA   0.14692    0.14766   0.995  0.32075

TG101209 0.1:RET51CA     0.02629    0.15813   0.166  0.86809

Zactima 0.25:RET51CA   -0.32952    0.17182  -1.918  0.05631 .

Zactima 0.5:RET51CA     -0.38473    0.16886  -2.278  0.02358 *

Zactima 1:RET51CA       -0.48314    0.20636  -2.341  0.02003 *
