# Supplementary material for: Ret function in muscle stem cells points to tyrosine kinase inhibitor therapy for facioscapulohumeral muscular dystrophy
Source: eLife. 2016 Nov 14;5:e11405. doi: 10.7554/eLife.11405 (PMC5108591; doi:10.7554/eLife.11405)
Supplement: Figure 9—Source data 1. — (a) Maximum likelihood parameters for a logistic model containing an interaction term, and a random effect term (the mouse) that describes the proportion of myoblasts incorporating EdU transduced with DUX4 or control (MIG) retrovirus when exposed to Sunitinib or DMSO. y represents the probability of EdU incorporation. µ represents the intercept parameter (representing the control treatment: MIG control retrovirus with no drug), β are the parameters representing the effects of each treatment, or the interaction as specified and δ indicates whether the effect is present or absent. (b) Corresponding log of odds ratios computed from the model, for all 4 tested conditions. DOI: http://dx.doi.org/10.7554/eLife.11405.014 [file elife-11405-fig9-data1.docx]

**Figure 9: Supplemental Table 1**

(a) Maximum likelihood parameters for a logistic model containing an interaction term, and a random effect term (the mouse) that describes the proportion of cells incorporating EdU infected with DUX4 or MIG control retrovirus when exposed to Sunitinib or DMSO. *y* represents the probability of EdU incorporation. µ represents the intercept parameter (representing the control treatment: MIG control retrovirus with no drug), *β* are the parameters representing the effects of each treatment, or the interaction as specified and δ indicates whether the effect is present or absent. (b) Corresponding log of odds ratios computed from the model, for all 4 tested conditions.

                        Estimate Std. Error z value Pr(>|z|)

Intercept         -0.265960   0.070142  -3.792  0.00015 ***

DUX4               -2.100246   0.128804 -16.306  < 2e-16 ***

Sunitinib      -0.002661   0.071947  -0.037  0.97050

DUX4 and Sunitinib 0.010464   0.181391   0.058  0.95400

Treatment Ratio Low C.I. High C.I.

CONTROL:DMSO             0.4332   0.3995    0.4676

DMSO:Sunitinib 0.4339  0.4005    0.4679

DUX4:Sunitinib 0.0864  0.0687    0.1081

DUX4:Sunitinib 0.0858   0.0680    0.1076
