# Supplementary material for: Ret function in muscle stem cells points to tyrosine kinase inhibitor therapy for facioscapulohumeral muscular dystrophy
Source: eLife. 2016 Nov 14;5:e11405. doi: 10.7554/eLife.11405 (PMC5108591; doi:10.7554/eLife.11405)
Supplement: Figure 9—Source data 2. — (a) Maximum likelihood parameters for a logistic model containing an interaction term, and a random effect term (the mouse) that describes the proportion of cells expressing MyoD transduced with DUX4 or MIG control retrovirus when exposed to Sunitinib or DMSO. y represents the probability of MyoD expression. µ represents the intercept parameter (representing the control treatment: MIG control retrovirus with no drug), β are the parameters representing the effects of each treatment, or the interaction as specified and δ indicates whether the effect is present or absent. (b) Corresponding log of odds ratios computed from the model, for all 4 tested conditions. DOI: http://dx.doi.org/10.7554/eLife.11405.015 [file elife-11405-fig9-data2.docx]

**Figure 9: Supplemental Table 2**

(a) Maximum likelihood parameters for a logistic model containing an interaction term, and a random effect term (the mouse) that describes the proportion of cells expressing MyoD infected with DUX4 or MIG control retrovirus when exposed to Sunitinib or DMSO. *y* represents the probability of MyoD expression. µ represents the intercept parameter (representing the control treatment: MIG control retrovirus with no drug), *β* are the parameters representing the effects of each treatment, or the interaction as specified and δ indicates whether the effect is present or absent. (b) Corresponding log of odds ratios computed from the model, for all 4 tested conditions.

                       Estimate Std. Error z value Pr(>|z|)

(Intercept)             1.81197    0.13145  13.785  < 2e-16 ***

DUX4           -3.99170    0.13504 -29.560  < 2e-16 ***

Sunitinib           -0.08889    0.10816  -0.822 0.411200

DUX4 and Sunitinib 0.63351    0.17816   3.556 0.000377 ***

Treatment Ratio Low C.I. High C.I.

CONTROL:DMSO 0.8596   0.8255   0.8879

DMSO:Sunitinib 0.8485   0.8129   0.8784

DUX4:Sunitinib 0.1016   0.0775   0.1321

DUX4:Sunitinib 0.1631   0.1293   0.2037
