# Supplementary material for: Ret function in muscle stem cells points to tyrosine kinase inhibitor therapy for facioscapulohumeral muscular dystrophy
Source: eLife. 2016 Nov 14;5:e11405. doi: 10.7554/eLife.11405 (PMC5108591; doi:10.7554/eLife.11405)
Supplement: Figure 10—source data 1. — (a) Maximum likelihood parameters for a logistic model containing an interaction term between DUX4 expression and Sunitinib during fusion of satellite-cells grown at high density and transduced with DUX4-expressing retrovirus or control (MIG). y represents the log-of-odds of the fusion index. µ represents the intercept parameter (representing the control treatment: no retrovirus, with no drug present), β are the parameters representing the effects of each treatment, or the interaction as specified and δ indicates whether the effect is present or absent. (b) Corresponding log of odds ratios computed from the model for all 4 tested conditions. DOI: http://dx.doi.org/10.7554/eLife.11405.017 [file elife-11405-fig10-data1.docx]

**Figure 10: Supplemental Table 1**

(a) Maximum likelihood parameters for a logistic model containing an interaction term between DUX4 expression and Sunitinib during fusion of satellite-cells grown at high density and infected or uninfected with DUX4-expressing retrovirus. *y* represents the log-of-odds of the fusion index. µ represents the intercept parameter (representing the control treatment: no retrovirus, with no drug present), *β* are the parameters representing the effects of each treatment, or the interaction as specified and δ indicates whether the effect is present or absent. (b) Corresponding log of odds ratios computed from the model for all 4 tested conditions.

a)

Parameter   Estimate  Std.err.   z value   P value

Intercept      0.105    0.0397     2.654   0.00794

DUX4          -0.901    0.0814   -11.065   < 2e-16

Sunitinib      0.024    0.0434     0.563     0.574

Interaction    1.767    0.0869    20.324   < 2e-16

b)

Treatment        Ratio  Low C.I. High C.I.

CONTROL:DMSO     0.526     0.507     0.546

DUX4:DMSO         0.311     0.282     0.342

CONTROL:SUNITINIB 0.532     0.524     0.541

DUX4:SUNITINIB    0.730     0.720     0.740
