# Supplementary material for: Ret function in muscle stem cells points to tyrosine kinase inhibitor therapy for facioscapulohumeral muscular dystrophy
Source: eLife. 2016 Nov 14;5:e11405. doi: 10.7554/eLife.11405 (PMC5108591; doi:10.7554/eLife.11405)
Supplement: Figure 10—source data 2. — (a) Maximum likelihood parameters for a logistic model containing an interaction term, and a random effect term (the mouse) that describes the fusion index of cells infected with DUX4 or MIG control retrovirus and grown at high density when exposed to Sunitinib or DMSO. y represents the log-of-odds of the fusion index. µ represents the intercept parameter (representing the control treatment: MIG control retrovirus with no drug), β are the parameters representing the effects of each treatment, or the interaction as specified and δ indicates whether the effect is present or absent. (b) Corresponding log of odds ratios computed from the model, for all 4 tested conditions. DOI: http://dx.doi.org/10.7554/eLife.11405.018 [file elife-11405-fig10-data2.docx]

**Figure 10: Supplemental Table 2**

(a) Maximum likelihood parameters for a logistic model containing an interaction term, and a random effect term (the mouse) that describes the fusion index of cells infected with DUX4 or MIG control retrovirus and grown at high density when exposed to Sunitinib or DMSO. *y* represents the log-of-odds of the fusion index. µ represents the intercept parameter (representing the control treatment: MIG control retrovirus with no drug), *β* are the parameters representing the effects of each treatment, or the interaction as specified and δ indicates whether the effect is present or absent. (b) Corresponding log of odds ratios computed from the model, for all 4 tested conditions.

a)

 Parameter                   Estimate Std. Error z value Pr(>|z|)

(Intercept)                  1.04035    0.04454  23.358   <2e-16 ***

DUX4                        -2.03527    0.07349 -27.694   <2e-16 ***

SUNITINIB                    0.13110    0.05146   2.547   0.0108 *

Interaction                  1.56783    0.09772  16.045   <2e-16 ***

*Recovery                    -0.33633    0.06507 -5.1685 2.36e-07 ****

b)

Treatment         Low C.I. Ratio Estimate High C.I.

CONTROL:DMSO         0.722          0.739     0.755

CONTROL:SUNITINIB    0.748          0.763     0.779

DUX4:DMSO            0.244          0.270     0.297

DUX4:SUNITINIB       0.643          0.669     0.694
