# Supplementary material for: Ret function in muscle stem cells points to tyrosine kinase inhibitor therapy for facioscapulohumeral muscular dystrophy
Source: eLife. 2016 Nov 14;5:e11405. doi: 10.7554/eLife.11405 (PMC5108591; doi:10.7554/eLife.11405)
Supplement: Figure 10—source data 3. — (a) Maximum likelihood parameters for a logistic model containing an interaction term, and a random effect term (the mouse) to describe MyoG expression in SCs exposed to Sunitinib or DMSO and expressing DUX4 or control (MIG) retrovirus at 1 or 2 days of culture when grown at low density. y represents the probability of MyoG expression. µ represents the intercept parameter (representing the control treatment: MIG control retrovirus with no drug), β are the parameters representing the effects of each treatment (e.g. β1 at day 1, β2 at day 2), or the interaction as specified and δ indicates whether the effect is present or absent. (b) Corresponding ratios computed from the model, for all 4 tested conditions. DOI: http://dx.doi.org/10.7554/eLife.11405.019 [file elife-11405-fig10-data3.docx]

**Figure 10: Supplemental Table 3**

(a) Maximum likelihood parameters for a logistic model containing an interaction term, and a random effect term (the mouse) to describe MyoG expression in SCs exposed to Sunitinib or DMSO and expressing DUX4 or MIG control retrovirus at 1 or 2 days of culture when grown at low density. *y* represents the probability of MyoG expression. µ represents the intercept parameter (representing the control treatment: MIG control retrovirus with no drug), *β* are the parameters representing the effects of each treatment (e.g. β_1_ at day 1, β_2_ at day 2), or the interaction as specified and δ indicates whether the effect is present or absent. (b) Corresponding ratios computed from the model, for all 4 tested conditions.

Parameter                            Estimate Std. Error z value Pr(>|z|)

(Intercept)                  -0.976225   0.095577 -10.214  < 2e-16 ***

Days 2                        1.685459   0.101046  16.680  < 2e-16 ***

Days 1:DUX4               -1.114718   0.128838  -8.652  < 2e-16 ***

Days 2:DUX4               -2.969503   0.132331 -22.440  < 2e-16 ***

Days 1:Sunitinib           0.005883   0.104327   0.056    0.955

Days 2:Sunitinib           -0.133411   0.096388  -1.384    0.166

Days 1:DUX4:Sunitinib   0.746182   0.169145   4.411 1.03e-05 ***

Days 2:DUX4:Sunitinib   0.752495   0.173101   4.347 1.38e-05 ***

Day DUX4:Sunitinib Ratio Low C.I. High C.I.

1 Control + DMSO 0.2736   0.2380          0.3124

1 Control + Sunitinib 0.2748   0.2396          0.3131

1 DUX4 + DMSO 0.1100   0.0888          0.1354

1 DUX4 + Sunitinib 0.2077   0.1769          0.2423

2 Control + DMSO 0.6702   0.6299          0.7082

2 Control + Sunitinib 0.6401   0.5982          0.6800

2 DUX4 + DMSO 0.0945   0.0751          0.1183

2 DUX4 + Sunitinib 0.1623   0.1358          0.1928
