# Supplementary material for: Ret function in muscle stem cells points to tyrosine kinase inhibitor therapy for facioscapulohumeral muscular dystrophy
Source: eLife. 2016 Nov 14;5:e11405. doi: 10.7554/eLife.11405 (PMC5108591; doi:10.7554/eLife.11405)
Supplement: Figure 10—source data 4. — (a) Maximum likelihood parameters for a logistic model containing an interaction term, and a random effect term (the mouse) to describe the MyHC expression in SCs exposed to Sunitinib or DMSO and expressing DUX4 or control (MIG) retrovirus at 1 or 2 days of culture when grown at low density. y represents the probability of MyHC expression. µ represents the intercept parameter (representing the control treatment: MIG control retrovirus with no drug), β are the parameters representing the effects of each treatment (e.g. β1 at day 1, β2 at day 2), or the interaction as specified and δ indicates whether the effect is present or absent. (b) Corresponding ratios computed from the model, for all 4 tested conditions. DOI: http://dx.doi.org/10.7554/eLife.11405.020 [file elife-11405-fig10-data4.docx]

**Figure 10: Supplemental Table 4**

(a) Maximum likelihood parameters for a logistic model containing an interaction term, and a random effect term (the mouse) to describe MyHC expression in SCs exposed to Sunitinib or DMSO and expressing DUX4 or MIG control retrovirus at 1 or 2 days of culture when grown at low density. *y* represents the probability of MyHC expression. µ represents the intercept parameter (representing the control treatment: MIG control retrovirus with no drug), *β* are the parameters representing the effects of each treatment (e.g. β_1_ at day 1, β_2_ at day 2), or the interaction as specified and δ indicates whether the effect is present or absent. (b) Corresponding ratios computed from the model, for all 4 tested conditions.

a)

Parameter                Estimate Std. Error z value Pr(>|z|)

(Intercept)              -1.42432    0.10827 -13.156  < 2e-16 ***

Days 2                     1.38906    0.10641  13.054  < 2e-16 ***

Days 1:DUX4               -2.27886    0.23129  -9.853  < 2e-16 ***

Days 2:DUX4               -3.16428    0.18259 -17.330  < 2e-16 ***

Days 1:Sunitinib           0.10749    0.11549   0.931  0.35203

Days 2:Sunitinib           0.05296    0.09415   0.563  0.57373

Days 1:DUX4:Sunitinib  1.12318    0.27425   4.096 4.21e-05 ***

Days 2:DUX4:Sunitinib   0.61641    0.23220   2.655  0.00794 **

b)

Day DUX4:Sunitinib Ratio  Low C.I. High C.I.

1 control + DMSO 0.1940   0.1629         0.2293

1 control + Sunitinib 0.2113   0.1788         0.2480

1 DUX4 + DMSO 0.0241   0.0156         0.0370

1 DUX4 + Sunitinib 0.0778   0.0600         0.1002

2 control + DMSO 0.4912   0.4444         0.5381

2 control + Sunitinib 0.5044   0.4574         0.5514

2 DUX4 + DMSO 0.0392   0.0277         0.0552

2 DUX4 + Sunitinib 0.0738   0.0566         0.0956
