# Supplementary material for: Ret function in muscle stem cells points to tyrosine kinase inhibitor therapy for facioscapulohumeral muscular dystrophy
Source: eLife. 2016 Nov 14;5:e11405. doi: 10.7554/eLife.11405 (PMC5108591; doi:10.7554/eLife.11405)
Supplement: Figure 11—source data 1. — The model is a linear model that describes the relationship between the shape (eccentricity) of 54.6 (control) and 54.12 (FSHD) cells relative to different doses of Sunitinib. P values are approximate and are based on the t-value and represent the probability that there is a difference in cell shape at a specific concentration of Sunitnib. y represents cell eccentricity, µ represents the intercept parameter (representing the control treatment: 54.6 cells with no drug), β are the parameters representing the effects of each treatment, or the interaction as specified and δ indicates whether the effect is present or absent. DOI: http://dx.doi.org/10.7554/eLife.11405.022 [file elife-11405-fig11-data1.docx]

**Figure 11: Supplementary Table 1**

Maximum likelihood parameters for a linear model containing an interaction term between the cell line and Sunitinib and incorporating a random effect term (the experiment). The model is a linear model that describes the relationship between the shape (eccentricity) of 54.6 (control) and 54.12 (FSHD) cells relative to different doses of Sunitinib. P values are approximate and are based on the t-value and represent the probability that there is a difference in cell shape at a specific concentration of Sunitnib. *y* represents cell eccentricity, µ represents the intercept parameter (representing the control treatment: 54.6 cells with no drug), *β* are the parameters representing the effects of each treatment, or the interaction as specified and δ indicates whether the effect is present or absent.

                       Estimate Std. Error t value Pr(>|t|)

(Intercept)             0.8060526  0.0076921  104.79  < 2e-16 ***

Sunitinib 125  0.0007486  0.0061040    0.12  0.90261

Sunitinib 250 -0.0063433  0.0061040   -1.04  0.30099

Sunitinib 500  0.0065669  0.0061040    1.08  0.28435

Sunitinib 750 -0.0021753  0.0061040   -0.36  0.72224

FSHD     0.0193053  0.0061040    3.16  0.00202 **

Sunitinib 125:FSHD   0.0189015  0.0058850    3.21  0.00179 **

Sunitinib 250:FSHD 0.0196323  0.0059881    3.28  0.00138 **

Sunitinib 500:FSHD 0.0016575  0.0061040    0.27  0.78648

Sunitinib 750:FSHD   0.0102796  0.0061040    1.68  0.09500 .
