# Supplementary material for: Ret function in muscle stem cells points to tyrosine kinase inhibitor therapy for facioscapulohumeral muscular dystrophy
Source: eLife. 2016 Nov 14;5:e11405. doi: 10.7554/eLife.11405 (PMC5108591; doi:10.7554/eLife.11405)
Supplement: Figure 11—source data 2. — (a) Maximum likelihood parameters for a logistic model containing an interaction term between the cell line and sunitinib and incorporating a random effect term (the experiment). The model is a binomial model that tests the relationship between the proliferation of 54.6 (control) and 54.12 (FSHD) cells relative to different doses of Sunitinib. P values represent the probability of a difference in proliferation between the control cells with varying doses of Sunitinib and between control and FSHD cells at different doses of Sunitinib. y represents the proliferation index. µ represents the intercept parameter (representing the control treatment: 54.6 cells with no drug), β are the parameters representing the effects of each treatment, or the interaction as specified and δ indicates whether the effect is present or absent. (b) Corresponding log of odds ratios computed from the model, for all 4 tested conditions. DOI: http://dx.doi.org/10.7554/eLife.11405.023 [file elife-11405-fig11-data2.docx]

**Figure 11: Supplementary Table 2**

a) Maximum likelihood parameters for a logistic model containing an interaction term between the cell line and sunitinib and incorporating a random effect term (the experiment). The model is a binomial model that tests the relationship between the proliferation of 54.6 (control) and 54.12 (FSHD) cells relative to different doses of Sunitinib. P values represent the probability of a difference in proliferation between the control cells with varying doses of Sunitinib and between control and FSHD cells at different doses of Sunitinib. *y* represents the proliferation index. µ represents the intercept parameter (representing the control treatment: 54.6 cells with no drug), *β* are the parameters representing the effects of each treatment, or the interaction as specified and δ indicates whether the effect is present or absent. (b) Corresponding log of odds ratios computed from the model, for all 4 tested conditions.

a)

                       Estimate Std. Error z value Pr(>|z|)

(Intercept)            -0.42241    0.04249  -9.942  < 2e-16 ***

Sunitinib 125           0.16640    0.06193   2.687 0.007212 **

Sunitinib 250           0.09586    0.05881   1.630 0.103107

Sunitinib 500          -0.04183    0.06189  -0.676 0.499161

Sunitinib 750          -0.08055    0.05961  -1.351 0.176628

FSHD   -0.28239    0.06706  -4.211 2.55e-05 ***

Sunitinib 125:FSHD -0.23422    0.06669  -3.512 0.000445 ***

Sunitinib 250:FSHD -0.05570    0.06459  -0.862 0.388491

Sunitinib 500:FSHD -0.01153    0.07004  -0.165 0.869297

Sunitinib 750:FSHD -0.10628    0.06835  -1.555 0.119972

b)

Parameters Low C.I. Ratio Estimate High C.I.

Sunitinib 0:control   0.376          0.396     0.416

Sunitinib 125:control 0.415          0.436     0.458

Sunitinib 250:control     0.400          0.419     0.439

Sunitinib 500:control     0.365          0.386     0.407

Sunitinib 750:control     0.358          0.377     0.396

Sunitinib 0:FSHD 0.309         0.331     0.354

Sunitinib 125:FSHD 0.357          0.380     0.403

Sunitinib 250:FSHD    0.382          0.406     0.430

Sunitinib 500:FSHD         0.359          0.383     0.408

Sunitinib 750:FSHD     0.328          0.352     0.377
