# Supplementary material for: Ret function in muscle stem cells points to tyrosine kinase inhibitor therapy for facioscapulohumeral muscular dystrophy
Source: eLife. 2016 Nov 14;5:e11405. doi: 10.7554/eLife.11405 (PMC5108591; doi:10.7554/eLife.11405)
Supplement: Figure 11—source data 3. — (a) Maximum likelihood parameters for a logistic model containing an interaction term between the cell line and Sunitinib and incorporating a random effect term (the experiment). The model is a binomial model that tests the relationship between the fusion of 54.6 (control) and 54.12 (FSHD) cells relative to different doses of Sunitinib. Estimate represents the relative change in fusion between conditions. p values represent the probability of a difference in fusion between the control cells with varying doses of Sunitinib and between control and FSHD cells at different doses of Sunitinib. y represents the log-of-odds of the fusion index. µ represents the intercept parameter (representing the control treatment: 54.6 cells with no drug), β are the parameters representing the effects of each treatment, or the interaction as specified and δ indicates whether the effect is present or absent. (b) Corresponding log of odds ratios computed from the model, for all 4 tested conditions. DOI: http://dx.doi.org/10.7554/eLife.11405.024 [file elife-11405-fig11-data3.docx]

**Figure 11: Supplementary Table 3**

a) Maximum likelihood parameters for a logistic model containing an interaction term between the cell line and Sunitinib and incorporating a random effect term (the experiment). The model is a binomial model that tests the relationship between the fusion of 54.6 (control) and 54.12 (FSHD) cells relative to different doses of Sunitinib. Estimate represents the relative change in fusion between conditions. P values represent the probability of a difference in fusion between the control cells with varying doses of Sunitinib and between control and FSHD cells at different doses of Sunitinib. *y* represents the log-of-odds of the fusion index. µ represents the intercept parameter (representing the control treatment: 54.6 cells with no drug), *β* are the parameters representing the effects of each treatment, or the interaction as specified and δ indicates whether the effect is present or absent. (b) Corresponding log of odds ratios computed from the model, for all 4 tested conditions.

a)

                       Estimate Std. Error z value Pr(>|z|)

(Intercept)             0.74077    0.09022    8.21  < 2e-16 ***

Sunitinib 125           0.14834    0.02892    5.13 2.91e-07 ***

Sunitinib 250           0.03045    0.02857    1.07    0.287

Sunitinib 500           0.43074    0.03167   13.60  < 2e-16 ***

Sunitinib 750           0.36837    0.02997   12.29  < 2e-16 ***

FSHD   -1.85964    0.03452  -53.88  < 2e-16 ***

Sunitinib 125:FSHD -1.07835    0.03288  -32.79  < 2e-16 ***

Sunitinib 250:FSHD -0.37114    0.03292  -11.27  < 2e-16 ***

Sunitinib 500:FSHD -1.09843    0.03513  -31.26  < 2e-16 ***

Sunitinib 750:FSHD -1.34316    0.03494  -38.44  < 2e-16 ***

b)

Sunitinib:FSHD Low C.I. Ratio Estimate High C.I.

0:FALSE           0.637          0.677     0.715

125:FALSE         0.671          0.709     0.744

250:FALSE         0.644          0.684     0.721

500:FALSE         0.730          0.763     0.794

750:FALSE         0.717          0.752     0.784

0:TRUE            0.214          0.246     0.281

125:TRUE          0.409          0.453     0.498

250:TRUE          0.555          0.599     0.641

500:TRUE          0.473          0.518     0.563

750:TRUE          0.398          0.442     0.487
