# Supplementary material for: Ret function in muscle stem cells points to tyrosine kinase inhibitor therapy for facioscapulohumeral muscular dystrophy
Source: eLife. 2016 Nov 14;5:e11405. doi: 10.7554/eLife.11405 (PMC5108591; doi:10.7554/eLife.11405)
Supplement: Figure 12—source data 1. — (a) Maximum likelihood parameters for a logistic model containing a random effect term (the mouse) describing the probability of a human LAMIN A/C+ nuclei being present in a murine muscle fibre. (b) Corresponding ratios computed from the model for 2 conditions with the ratio representing the probability of a LAMIN A/C+ nuclei occurring in a muscle fibre. In all condition but the control (Intercept), the error contribution of the baseline (Intercept) has been omitted when computing the confidence intervals (C.I.). y represents the log-of-odds of the LAMIN A/C+ nuclei occurring in a muscle fibre relative to all LAMIN A/C+ nuclei. µ represents the intercept parameter (representing the control treatment: PBS), β are the parameters representing the effects of each treatment and δ indicates whether the effect is present or absent. DOI: http://dx.doi.org/10.7554/eLife.11405.026 [file elife-11405-fig12-data1.docx]

**Figure 12: Supplementary Table 1**

Binomial model evaluating whether the proportion of Lamin A/C+ nuclei in a muscle fibre is significantly affected by exposure to Sunitinib.

(a) Maximum likelihood parameters for a logistic model containing a random effect term (the mouse) describing the probability of a Lamin A/C+ nuclei being present in a muscle fibre. (b) Corresponding ratios computed from the model for 2 conditions with the ratio representing the probability of a Lamin A/C+ nuclei occurring in a fibre. In all condition but the control (Intercept), the error contribution of the baseline (Intercept) has been omitted when computing the confidence intervals (C.I.). *y* represents the log-of-odds of the Lamin A/C+ nuclei occurring in a fibre relative to all Lamin A/C+ nuclei. µ represents the intercept parameter (representing the control treatment: PBS), *β* are the parameters representing the effects of each treatment and δ indicates whether the effect is present or absent.

a)

             Estimate Std. Error z value Pr(>|z|)

(Intercept)   0.43326    0.02415  17.942   <2e-16 ***

Sunitinib  0.25497    0.03044   8.377   <2e-16 ***

b)          Ratio   Low C.I.  High C.I.

Control     0.607   0.595   0.618

Sunitinib   0.666   0.657   0.674
