# Supplementary material for: Ret function in muscle stem cells points to tyrosine kinase inhibitor therapy for facioscapulohumeral muscular dystrophy
Source: eLife. 2016 Nov 14;5:e11405. doi: 10.7554/eLife.11405 (PMC5108591; doi:10.7554/eLife.11405)
Supplement: Figure 12—source data 2. — (a) Maximum likelihood parameters for a logistic model containing a random effect term (the mouse) describing the probability of a SPECTRIN+ murine muscle fibre containing LAMIN A/C+ nuclei . (b) Corresponding ratios computed from the model for 2 conditions with the ratio representing the probability of a SPECTRIN+ murine muscle fibre containing LAMIN A/C+ nuclei. In all condition but the control (Intercept), the error contribution of the baseline (Intercept) has been omitted when computing the confidence intervals (C.I.). y represents the log-of-odds of a SPECTRIN+ muscle fibre containing LAMIN A/C+ nuclei. µ represents the intercept parameter (representing the control treatment: PBS), β are the parameters representing the effects of each treatment and δ indicates whether the effect is present or absent. DOI: http://dx.doi.org/10.7554/eLife.11405.027 [file elife-11405-fig12-data2.docx]

**Figure 12: Supplementary Table 2**

Binomial model evaluating whether the proportion of muscle fibres showing Spectrin immunoreactivity and Lamin A/C nuclei is significantly affected by exposure to Sunitinib.

(a) Maximum likelihood parameters for a logistic model containing a random effect term (the mouse) describing the probability of a Spectrin+ fibre containing Lamin A/C+ nuclei. (b) Corresponding ratios computed from the model for 2 conditions with the ratio representing the probability of a Spectrin+ fibre containing Lamin A/C+ nuclei. In all condition but the control (Intercept), the error contribution of the baseline (Intercept) has been omitted when computing the confidence intervals (C.I.). *y* represents the log-of-odds of a Spectrin+ fibre containing Lamin A/C+ nuclei. µ represents the intercept parameter (representing the control treatment: PBS), *β* are the parameters representing the effects of each treatment and δ indicates whether the effect is present or absent.

             Estimate Std. Error z value Pr(>|z|)

(Intercept)  -1.50348    0.08085 -18.596  < 2e-16 ***

Sunitinib  0.42217    0.08929   4.728 2.26e-06 ***

            Ratio   Low C.I.  High C.I.

Control     0.160   0.182   0.207

Sunitinib   0.239   0.253   0.268
